# Performance Assessment of SARS-CoV-2 PCR Assays Developed by WHO Referral Laboratories

**DOI:** 10.3390/jcm9061871

**Published:** 2020-06-16

**Authors:** Sibyle Etievant, Antonin Bal, Vanessa Escuret, Karen Brengel-Pesce, Maude Bouscambert, Valérie Cheynet, Laurence Generenaz, Guy Oriol, Gregory Destras, Geneviève Billaud, Laurence Josset, Emilie Frobert, Florence Morfin, Alexandre Gaymard

**Affiliations:** 1 Laboratoire de Virologie, Institut des Agents Infectieux (IAI), Hospices Civils de Lyon, Groupement Hospitalier Nord, 69004 Lyon, France; antonin.bal@chu-lyon.fr (A.B.); vanessa.escuret@chu-lyon.fr (V.E.); maude.bouscambert-duchamp@chu-lyon.fr (M.B.); gregory.destras@chu-lyon.fr (G.D.); genevieve.billaud@chu-lyon.fr (G.B.); laurence.josset@chu-lyon.fr (L.J.); emilie.frobert@chu-lyon.fr (E.F.); florence.morfin-sherpa@chu-lyon.fr (F.M.); 2 Centre National de Référence des Virus Respiratoires, Hospices Civils de Lyon, Groupement Hospitalier Nord, 69004 Lyon, France; 3 Laboratoire Commun de Recherche Hospices Civils de Lyon–bioMérieux, Centre Hospitalier Lyon Sud, 69310 Pierre-Bénite, France; Karen.BRENGEL-PESCE@biomerieux.com (K.B.-P.); Valerie.CHEYNET@biomerieux.com (V.C.); laurence.generenaz@biomerieux.com (L.G.); Guy.ORIOL@biomerieux.com (G.O.); 4 Université de Lyon, Virpath, CIRI, INSERM U1111, CNRS UMR5308, ENS Lyon, Université Claude Bernard Lyon 1, F-69372 Lyon, France

**Keywords:** SARS-CoV-2, COVID-19, RT-PCR, diagnostics, sensitivity

## Abstract

A reliable diagnostic assay is crucial to early detect new COVID-19 cases and limit severe acute respiratory syndrome coronavirus 2 (SARS-CoV-2) transmission. Since the onset of the COVID-19 pandemic, the World Health Organization has published several diagnostic molecular approaches developed by referral laboratories, including Charité (Germany), HKU (Hong Kong), China CDC (China), US CDC (United States), and Institut Pasteur, Paris (France). We aimed to compare the sensitivity and specificity of these different RT-PCR assays using SARS-CoV-2 cell culture supernatants and clinical respiratory samples. Overall, the different RT-PCR assays performed well for SARS-CoV-2 detection and were all specific except the N Charité (Germany), and N2 US CDC (United States) assays. RdRp Institut Pasteur (IP2, IP4), N China CDC, and N1 US CDC were found to be the most sensitive assays. The data presented herein are of prime importance to facilitate the equipment choice of diagnostic laboratories, as well as for the development of marketed tests.

## 1. Introduction

A new human coronavirus called severe acute respiratory syndrome coronavirus 2 (SARS-CoV-2) emerged in China in December 2019 [1]. SARS-CoV-2 is responsible for coronavirus disease 2019 (COVID-19), which was declared a pandemic on 12 March 2020 by the World Health Organization (WHO) [2]. As of 26 May 2020, 5,404,512 cases have been reported, including 343,514 deaths [3]. A reliable diagnostic assay is crucial to limit the spread of SARS-CoV-2 as early detection of new cases leads to patient isolation and contact tracing. The first SARS-CoV-2 genome was published on 10 January 2020 [4], enabling the rapid design of a real-time reverse-transcriptase polymerase chain reaction (RT-PCR) assay by Charité (Germany) [5,6]. This test was the first to be dispatched by WHO [7] and was widely implemented in clinical virology laboratories worldwide [8]. Since then, WHO has published [9] other approaches developed by referral laboratories, including HKU (Hong Kong) [10,11], China CDC (China) [12], US CDC (United States) [13], and Institut Pasteur, Paris (France) [14]. These assays included several RT-PCRs targeting two or three different SARS-CoV-2 gene regions, including RdRp (RNA-dependent RNA polymerase), N (nucleocapsid protein), E (envelope protein), ORF1ab nsp10 (non-structural protein 10), and ORF1b nsp14 (non-structural protein 14). In the present study, we aimed to compare the sensitivity and specificity of these different RT-PCR assays. Overall, the different RT-PCR assays performed well for SARS-CoV-2 detection and were all specific except N Charité, (Germany) and N2 US CDC (United States) assays. RdRp Institut Pasteur (IP2, IP4), N China CDC, and N1 US CDC were found to be the most sensitive assays.

## 2. Materials and Methods

### 2.1. Study Design

To assess sensitivity, different RNA concentrations of SARS-CoV-2 cell culture supernatants, as well as clinical samples with different viral loads (*n* = 4), were tested using all RT-PCR assays. For the three most sensitive assays, limit of detection (LoD) was assessed, and additional clinical samples (*n* = 16) with low viral loads were also tested. To assess specificity, clinical samples negative for SARS-CoV-2 (*n* = 50) were tested using all RT-PCR assays. All clinical samples (nasopharyngeal aspirates) were provided by the Hospices Civils de Lyon—University Hospital, France, and frozen at 80 °C before extraction.

### 2.2. Sensitivity

The sensitivity for each RT-PCR assay was first assessed using ten-fold serial dilutions from 10^−3^ to 10^−9^ of SARS-CoV-2 cell culture supernatants (one replicate for 10^−3^ and 10^−4^, three replicates for 10^−5^ and 10^−6^, and five replicates for 10^−7^ to 10^−9^). Four positive clinical samples were then tested using all RT-PCR assays to confirm these results.

For the three most sensitive assays, we estimated the LoD using a probit analysis by including five additional replicates of each dilution of the cell culture supernatants. Probit analysis consists of describing the relationship between the probability of detection and concentration using a cumulative probability curve. For each dilution, the ratio (the hit rate) is computed as the number of replicates with a detected outcome per the total number of replicates tested. These hit rates are converted mathematically into cumulative normal probability units (probits) and fitted using a regression model vs. their respective concentrations. The LoD is defined as the lowest amount of viral genome that can be detected with a 95% hit rate. For these three most sensitive assays, the results obtained with probit analysis were confirmed by additional testing of sixteen clinical samples with low viral concentration.

### 2.3. Specificity

The specificity for each RT-PCR assay was assessed using clinical samples (*n* = 50) tested negative for SARS-CoV-2, including clinical samples (*n* = 30) tested positive for other respiratory viruses: human coronaviruses 229E, OC43, HKU1, and NL63, human influenza A and B viruses, rhinovirus, respiratory syncytial virus, parainfluenzavirus, adenovirus, metapneumovirus, and picornavirus.

Exploration of false-positive results was performed with additional negative clinical samples, water, and one additional clinical sample tested positive for each target. Amplicon size was analyzed using Agilent DNA 1000 kit (Agilent Technologies, Santa Clara, CA, USA).

### 2.4. SARS-CoV-2 Cell Culture Supernatants

Cell culture supernatants were obtained from a positive clinical sample cultivated in a biosafety level 3 laboratory on buffalo green monkey cells (cell line provided by the Université Louis Pasteur, Strasbourg, France) [15]. The SARS-CoV-2 culture had an infectious titer of 8.27 log_10_TCID_50_/mL as assessed by the Reed and Muench statistical method [16].

### 2.5. Extraction and RT-PCR

RNA extraction was performed using the EMAG^®^ platform (bioMérieux, Marcy-l’Étoile, France), according to manufacturer’s instructions. RT-PCR was performed following published instructions [5,6,10,11,12,13,14], which are summarized in Table 1; Table 2. Since the China CDC protocol does not specify polymerase, thermocycler, volume of nucleic acid extract, and amplification cycles, the same instructions as those for the HKU assay were applied. RdRp IP2 and IP4 assays from Institut Pasteur, Paris (France) can be multiplexed or used in simplex [14]. Preliminary comparison on SARS-CoV-2 cell culture supernatants found that RdRp IP4 performed better when used in multiplex, whereas IP2 was not significantly impacted (Supplementary Table S1). The CFX 96 Touch™ Real-Time PCR (Bio-Rad, Hercules, CA, USA) was used for all RT-PCR assays.

## 3. Results

### 3.1. Sensitivity Comparison of the Five RT-PCR Assays

The E Charité and N2 US CDC assays were positive for all specimens, including negative samples and negative controls (water). These false-positive results were explored (details below), but the sensitivity of these assays was not further assessed.

**Figure 1:**
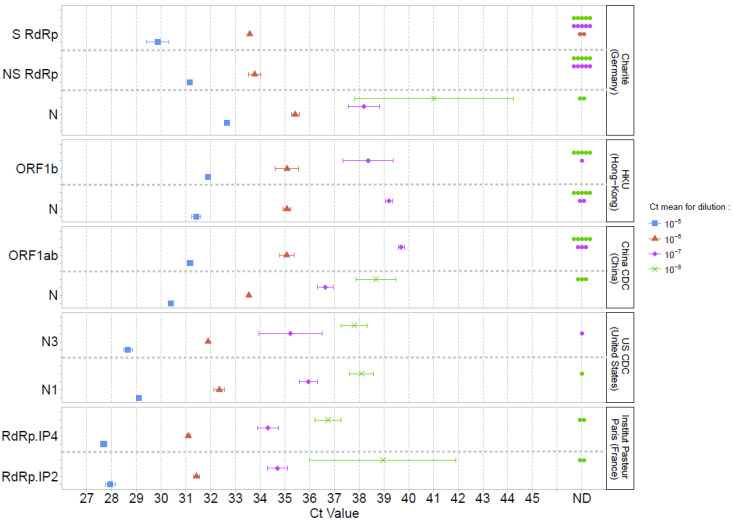
Mean Ct values and standard deviations obtained using five PCR-based methods for SARS-CoV-2 detection. Serial dilutions of SARS-CoV-2 cell culture supernatants were used and are represented by a single color (10^−5^ blue, 10^−6^ red, 10^−7^ pink, 10^−8^ green). A point in the ND (non-detected) column (Ct value axis) indicates a negative result for one replicate.

Sensitivity was first assessed using SARS-CoV-2 cell culture supernatants. Using both specific SARS-CoV-2 (S) and non-specific (NS; detecting SARS-CoV-2, SARS-CoV, and bat-SARS-related CoVs) RdRp Charité assays, all replicates of the 10^−5^ dilution (and inferior); 1/3 (S RdRp) and 3/3 (NS RdRp) of the 10^−6^ dilution replicates; and none of the 10^−7^, 10^−8^, and 10^−9^ dilutions (0/5) were detected. ORF1b and N HKU, and ORF1ab China CDC assays detected all replicates of dilutions inferior or equal to 10^−6^ and detected 4/5, 3/5, and 2/5 for 10^−7^ dilutions, respectively. None of these assays detected replicates of 10^−8^ (0/5) and 10^−9^ (0/5) dilutions. In contrast, N Charité, N China CDC, N1 and N3 US CDC, and duplex RdRp IP2/IP4 were positive for most replicates of the 10^−7^ (5/5, 5/5, 5/5, 4/5, 5/5 and 5/5, respectively) and 10^−8^ dilutions (3/5, 2/5, 4/5, 5/5, 3/5, 3/5, respectively; Figure 1). At 10^−9^ dilution, N China, N1 and N3 US CDC, and duplex RdRp IP2/IP4 assays were able to detect replicates (1/5, 1/5, 2/5, 3/5, 1/5, respectively).

The mean cycle threshold (Ct) values obtained for each assay were then compared for dilutions 10^−5^ to 10^−8^ (Figure 1, Supplementary Table S2). Since the accepted technical variability of RT-PCR is below 0.5 log_10_, we considered a difference of 2 Ct as significant [17]. At 10^−5^ dilution, the lowest Ct value was 27.7 for RdRp IP4. No significant difference in Ct values (Ct ranged from 28.0 to 29.1) was reported with N1 and N3 US CDC, and RdRp IP2. A similar Ct profile was observed for these assays at 10^−6^ and 10^−7^ dilutions. At 10^−8^ dilution, only N Charité had significantly higher Ct values (41.0 vs. 36.7 to 39.0 for N China CDC, N1 and N3 US CDC, and duplex RdRp IP2/IP4). 

Clinical samples (*n* = 4) were then tested using all RT-PCR assays to confirm the results obtained on SARS-CoV-2 cell culture supernatants (Supplementary Table S3). ORF1b and N HKU, ORF1ab and N China CDC, N1 and N3 US CDC, and RdRp IP2 and RdRp IP4 assays detected all four positive samples. S and NS RdRp, and N Charité assays did not detect the positive sample with the lowest viral concentration.

Taken together, N China CDC, N1 and N3 US CDC, as well as RdRp IP2 and IP4 were the most sensitive assays.

### 3.2. Limit of Detection for the Most Sensitive Assays

Due to a limited quantity of clinical sample available, only one target from each referral laboratory providing the most sensitive assays was tested: N China CDC, N1 US CDC, and RdRp IP2. The LoD of N3 US CDC was not determined as this assay is not specific for SARS-CoV-2 detection and was removed from the new version of the US CDC assay [13]. We chose to determine the LoD for RdRp IP2 and not IP4 as IP2 detected more replicates at the 10^−9^ dilution on cell culture supernatants.

**Figure 2:**
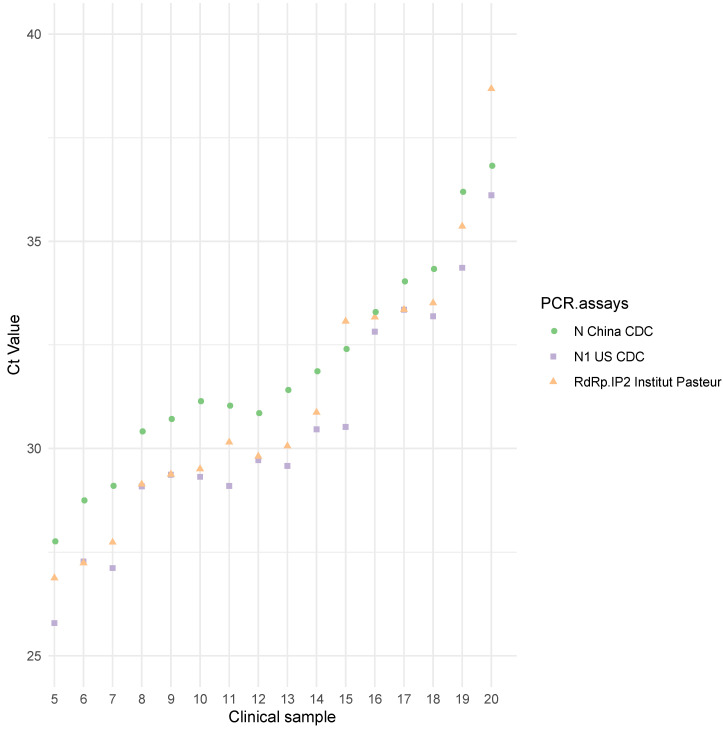
Ct values for 16 positive clinical samples using the three most sensitive assays. N China CDC is represented by green circles, N1 US CDC by purple squares, and RdRp IP2 Institut Pasteur, Paris, by orange triangles.

The 95% hit rate obtained was 1.36 log_10_TCID_50_/mL [0.8; 3.09] for N China CDC, 0.44 log_10_TCID_50_/mL [0.05; 1.83] for N1 US CDC, and 0.63 log_10_TCID_50_/mL [0.25; 1.9] for RdRp IP2. The differences observed were not statistically significant. For these three assays, the results were confirmed by additional testing of clinical samples (Figure 2, Supplementary Table S4). N1 US CDC and RdRp IP2 had lower Ct values than N China CDC (Figure 2), but no significant differences (Ct difference < 2) were observed.

### 3.3. Specificity 

No false-positive results were obtained on clinical samples that tested negative for SARS-CoV-2 and/or positive for other viruses than SARS-CoV-2, except for E Charité and N2 US CDC, which were positive for all specimens (Supplementary Table S5).

### 3.4. Exploration of E Charité and N2 US CDC False-Positive Results

Since E Charité and N2 US CDC assays were positive for all specimens and replicates, including negative samples and controls, false-positive results were further explored (Supplementary Figure S1).

For E Charité, negative samples showed two amplicons, one at 84 base pairs (bp) and one at 121 bp, whereas the positive sample only had one amplicon at 121 bp, which is close to the expected size of a specific amplification (Table 1). Thus, the false-positive amplification obtained using E Charité might be derived from a contamination (amplicon size at 121 bp) but could also be associated with an aspecific amplification (amplicon size at 84 bp). Using the N2 US CDC assay, negative samples showed one amplicon at 73 bp, which is close to the expected size of a specific amplification (Table 1). Thus, the false-positive amplification obtained using N2 US CDC might be due to a contamination. Sequencing of these amplicon products should be performed for further investigation.

## 4. Discussion

The present study compared the performance of five RT-PCR-based methods developed by referral laboratories. N China CDC, N1 US CDC, and RdRp IP2 and IP4 were found to be the most sensitive assays on SARS-CoV-2 cell culture supernatants and clinical respiratory samples. Vogels et al. compared the performance of SARS-CoV-2 PCR assays developed by the same referral laboratories, except those from Institut Pasteur. Using RNA-spiked mock samples, they found that ORF HKU was one of the most sensitive assays [18]. Herein, ORF HKU was more sensitive than RdRp Charité but slightly less sensitive than other assays, such as N1 US CDC or N China. Although RdRp Charité performed well for the lowest dilutions, it was nevertheless found to be less sensitive than others, a result in line with those of Vogels et al. [18]. It is worth noting that the Charité assay was the first to be published at the early stage of the pandemic [9] and has been widely used worldwide [8]. This assay was initially designed for the diagnosis of SARS-related CoVs and then optimized for SARS-CoV-2 detection [5]. Thanks to this assay, an important number of COVID-19 diagnoses were made, which contributed to limiting the spread of the outbreak. In line with the present results, it was reported that RdRp IP2 and RdRp IP4 sensitivity was similar when used in multiplex [14], suggesting that the Institut Pasteur assay should preferentially be used in multiplex. Of note, we did not apply the Ct cut-off values above, in which a sample would be considered negative, since such values were not provided in the protocols made available by the referral laboratories. 

As previously reported [19], we identified probable primer contamination using N2 US CDC and E Charité, which prevented us from further evaluating their sensitivity and specificity. Except for these two assays, no false-positive results were observed when testing a wide range of respiratory viruses, a result consistent with previous studies [5,6,10,11,13,14]. Although not observed herein, the amplification of non-specific products for ORF1 and N China CDC, and N2 and N3 US CDC has also been reported [18]. 

The performance of other RT-PCR tests recently developed [7] should be explored in further studies. In addition, the quantification of SARS-CoV-2 could be performed to assess the effectiveness of potential treatments. The data presented herein are of prime importance to facilitate the equipment choice of all diagnostic laboratories, as well as for the development of marketed tests. Sensitive tests should be widely implemented to limit the spread of the current outbreak and prepare for the post-epidemic phase and future seasonal epidemics.

## Figures and Tables

**Table 1:** Summary of the five RT-PCR assays targeting severe acute respiratory syndrome coronavirus 2 (SARS-CoV-2).

Country (Institute)	Target	Oligonucleotide	Sequence	Amplicon Size ^a^	Polymerase	Thermocycler Used in the Reference Publication	Volume of RNA Extract
Charité (Germany) [5,6]	RdRp ^b^	Charité_RdRp_F	GTGARATGGTCATGTGTGGCGG	100 bp	SuperScript™ III Platinum^®^ One-Step Quantitative RT-PCR System	Light Cycler ^®^ 480II (Roche) or Applied Biosystems ViiA™7 (Therom Fisher)	5 µL
		Charité_S_RdRp_P ^c^	FAM-CAGGTGGAACCTCATCAGGAGATGC-BBQ				
		Charité_NS_RdRp_P ^d^	FAM-CCAGGTGGWACRTCATCMGGTGATGC-BBQ				
		Charité_RdRp_R	CARATGTTAAASACACTATTAGCATA				
	E ^e^	Charité_E_F	ACAGGTACGTTAATAGTTAATAGCGT	113 bp			
		Charité_E_P	FAM-ACACTAGCCATCCTTACTGCGCTTCG-BBQ				
		Charité_E_R	ATATTGCAGCAGTACGCACACA				
	N	Charité_N_F	CACATTGGCACCCGCAATC	128 bp			
		Charité_N_P	FAM-ACTTCCTCAAGGAACAACATTGCCA-BBQ				
		Charité_N_R	GAGGAACGAGAAGAGGCTTG				
HKU (Hong Kong) [10,11]	ORF1b-nsp14 ^f^	HKU_ORF_F	TGGGGYTTTACRGGTAACCT	132 bp	TaqMan Fast Virus Master mix	Applied Biosystems ViiA™7 (Therom Fisher)	4 µL
		HKU_ORF_P	FAM-TAGTTGTGATGCWATCATGACTAG-TAMRA				
		HKU_ORF_R	AACRCGCTTAACAAAGCACTC				
	N ^e^	HKU_N_F	TAATCAGACAAGGAACTGATTA	110 bp			
		HKU_N_P	FAM-GCAAATTGTGCAATTTGCGG-TAMRA				
		HKU_N_R	CGAAGGTGTGACTTCCATG				
China CDC (China) [12]	N	ChinaCDC_N_F	GGGGAACTTCTCCTGCTAGAAT	99 bp	Unspecified	Unspecified	Unspecified
		ChinaCDC_N_P	FAM-TTGCTGCTGCTTGACAGATT-TAMRA				
		ChinaCDC_N_R	CAGACATTTTGCTCTCAAGCTG				
	ORF1ab-nsp10	ChinaCDC_ORF_F	CCCTGTGGGTTTTACACTTAA	119 bp			
		ChinaCDC_ORF_P	FAM-CCGTCTGCGGTATGTGGAAAGGTTATGG-BHQ1				
		ChinaCDC_ORF_R	ACGATTGTGCATCAGCTGA				
US CDC (United States) [13]	N1 ^c^	USCDC_N1_F	GACCCCAAAATCAGCGAAAT	72 bp	TaqPath™ 1-Step RT-qPCR Master Mix, CG (Thermo Fisher)	Applied Biosystems™ 7500 Fast (Thermo Fisher)	5 µL
		USCDC_N1_P	FAM-ACCCCGCATTACGTTTGGTGGACC-BHQ1				
		USCDC_N1_R	TCTGGTTACTGCCAGTTGAATCTG				
	N2 ^c^	USCDC_N2_F	TTACAAACATTGGCCGCAAA	67 bp			
		USCDC_N2_P	FAM-ACAATTTGCCCCCAGCGCTTCAG-BHQ1				
		USCDC_N2_R	GCGCGACATTCCGAAGAA				
	N3 ^d^	USCDC_N3_F	GGGAGCCTTGAATACACCAAAA	72 bp			
		USCDC_N3_P	FAM-AYCACATTGGCACCCGCAATCCTG-BHQ1				
		USCDC_N3_R	TGTAGCACGATTGCAGCATTG				
Institut Pasteur, Paris (France) [14]	RdRp IP2 (Flo2)	Pasteur_IP2_F	ATGAGCTTAGTCCTGTTG	108 bp	SuperScript™ III Platinum^®^ One-Step Quantitative RT-PCR System	LightCycler ^®^ 480 (Roche)	5 µL
		Pasteur_IP2_P	HEX-AGATGTCTTGTGCTGCCGGTA-BHQ1				
		Pasteur_IP2_R	CTCCCTTTGTTGTGTTGT				
	RdRp IP4 (Flo4)	Pasteur_IP4_F	GGTAACTGGTATGATTTCG	107 bp			
		Pasteur_IP4_P	FAM-TCATACAAACCACGCCAGG-BHQ1				
		Pasteur_IP4_R	CTGGTCAAGGTTAATATAGG				

^a^ Amplicon size in base-pairs (bp) deduced from BetaCoV/Wuhan-Hu-1/2019 sequence (GISAID |EPI ISL 402125). ^b^ Target used for confirmation and discrimination of SARS-CoV-2 and SARS-CoV. ^c^ Probe specific for SARS-CoV-2. ^d^ Probe detecting SARS-CoV-2, SARS-CoV, and bat-SARS-related CoVs. ^e^ Target used for screening. ^f^ Target used for confirmation.

**Table 2:** Amplification cycles of the five RT-PCR assays targeting SARS-CoV-2.

Institute (Country)	Charité (Germany) [5,6]			HKU (Hong Kong) [10,11]			China CDC (China) [12]			US CDC (United States) [13]			Institut Pasteur, Paris (France) [14]		
Amplification Cycles	T °C	Time (min)	Number of Cycles	T °C	Time (min)	Number of Cycles	T°C	Time (min)	Number of Cycles	T °C	Time (min)	Number of Cycles	T °C	Time (min)	Number of Cycles
Uracil-N-glycosylase activation							Unspecified			25	02:00	1			
Reverse transcription	55	10:00	1	50	05:00	1				50	15:00		55	20:00	1
RT inactivation/Enzyme activation	95	03:00		95	00:20					95	02:00		95	03:00	
Denaturation	95	00:15	45	95	00:05	40				95	00:03	45	95	00:15	50
Annealing/Extending	58	00:30		60	00:30					55	00:30		58	00:30	
Cooling													40	00:30	1

## Supplementary Materials

The following are available online at https://www.mdpi.com/2077-0383/9/6/1871/s1. Figure S1: Electropherograms of amplicon sizes obtained using Agilent DNA 1000 kit (Agilent Technologies) for one positive sample and one negative sample for E Charité (Germany) and N2 US CDC (United States), respectively. Table S1: Cycle threshold values and mean ± standard deviation for serial dilutions of SARS-CoV-2 cell culture supernatants obtained using IP2 and IP4 Institut Pasteur, Paris (France) when used in simplex and multiplex. Table S2: Cycle threshold values and mean ± standard deviation for serial dilutions of SARS-CoV-2 cell culture supernatants obtained using five RT-PCR assays for SARS-CoV-2 detection made available by WHO, Table S3: Cycle thresholds values obtained on clinical samples (*n* = 4) using five RT-PCR assays for SARS-CoV-2 detection made available by WHO, Table S4: Cycle threshold values obtained on clinical samples (*n* = 16) using the three most sensitive methods: N China CDC, N1 US CDC, and IP2 Institut Pasteur, Paris (France). Table S5: Specificity assessment of five RT-PCR assays made available by WHO.

## References

[1] (2020). Coronaviridae Study Group of the International Committee on Taxonomy of Viruses The species Severe acute respiratory syndrome related coronavirus: Classifying 2019-nCoV and naming it SARS-CoV-2. Nat. Microbiol..

[2] World Health Organization WHO Announces COVID-19 Outbreak a Pandemic. http://www.euro.who.int/en/health-topics/health-emergencies/coronavirus-covid-19/news/news/2020/3/who-announces-covid-19-outbreak-a-pandemic.

[3] World Health Organization Coronavirus Disease 2019 (COVID-19)—Situation Report—127. https://www.who.int/docs/default-source/coronaviruse/situation-reports/20200416-sitrep-87-covid-19.pdf?sfvrsn=9523115a_2.

[4] Holmes E.C. Novel 2019 Coronavirus Genome. http://virological.org/t/novel-2019-coronavirus-genome/319.

[5] Corman V., Landt O., Kaiser M., Molenkamp R., Meijer A., Chu D., Bleicker T., Brünink S., Schneider J., Schmidt M.L. (2020). Detection of 2019 novel coronavirus (2019-nCoV) by real-time RT-PCR. Eurosurveillance.

[6] Corman V., Bleicker T., Brünink S., Drosten C. Diagnostic Detection of 2019-nCoV by Real-Time RT-PCR; Charité Virology, Berlin, Germany. https://www.who.int/docs/default-source/coronaviruse/protocol-v2-1.pdf?sfvrsn=a9ef618c_2.

[7] Cormac S. Coronavirus and the Race to Distribute Reliable Diagnostics. https://www.nature.com/articles/d41587-020-00002-2.

[8] Reusken C., Broberg E., Haagmans B., Meijer A., Corman V., Papa A., Charrel R., Drosten C., Koopmans M., Leitmeyer K. (2020). Laboratory readiness and response for novel coronavirus (2019-nCoV) in expert laboratories in 30 EU/EEA countries, January 2020. Eurosurveillance.

[9] World Health Organization Coronavirus Disease (COVID-19) Technical Guidance: Laboratory Testing for 2019-nCoV in Humans. https://www.who.int/emergencies/diseases/novel-coronavirus-2019/technical-guidance/laboratory-guidance.

[10] Chu D., Pan Y., Cheng S., Hui K., Krishnan P., Liu Y., Ng D., Wan C., Yang P., Wang Q. (2020). Molecular diagnosis of a novel Coronavirus (2019-nCoV) causing an outbreak of pneumonia. Clin. Chem..

[11] HKU Med Detection of 2019 Novel Coronavirus (2019-nCoV) in Suspected Human Cases by RT-PCR. https://www.who.int/docs/default-source/coronaviruse/peiris-protocol-16-1-20.pdf?sfvrsn=af1aac73_4.

[12] China CDC China CDC Primers and Probes for Detection 2019-nCoV. http://ivdc.chinacdc.cn/kyjz/202001/t20200121_211337.html.

[13] Centers for Disease Control and Prevention A CDC 2019-Novel Coronavirus (2019-nCoV) Real-Time RT-PCR Diagnostic Panel. https://www.fda.gov/media/134922/download.

[14] Institut Pasteur, Paris Protocol: Real-Time RT-PCR Assays for the Detection of SARS-CoV-2. https://www.who.int/docs/default-source/coronaviruse/real-time-rt-pcr-assays-for-the-detection-of-sars-cov-2-institut-pasteur-paris.pdf?sfvrsn=3662fcb6_2.

[15] Barron A., Olshevsky C., Cohen M. (1970). Characteristics of the BGM line of cells from African green monkey kidney. Brief report. Arch. Gesamte Virusforsch..

[16] Reed L., Muench H. (1938). A Simple Method of Estimating Fifty Per Cent Endpoints. Am. J. Epidemiol..

[17] Baraduc M., Baume A., Bouvet F., Canis C., Cattoen S., Charachon V., Cocquerelle R., Courcol C., de Champs M., Ferroni N. (2014). SFM comparaison de methodes. Comite Qualite (QUAMIC) de la Société Francaise de Microbiologie—Recommandations 2014.

[18] Vogels C., Brito A., Wyllie A., Fauver J., Ott I., Kalinich C., Petrone M., Landry M., Foxman E., Grubaugh N. Analytical sensitivity and efficiency comparisons of SARS-COV-2 qRT-PCR assays (Preprint). Prepr. MedRxiv..

[19] Mögling R., Meijer A., Berginc N., Bruisten S., Charrel R., Coutard B., Eckerle I., Enouf V., Hungnes O., Korukluoglu G. (2020). Early release—Delayed laboratory response to COVID-19 caused by molecular diagnostic contamination. Emerg. Infect. Dis. J. CDC..

