# Supplementary material for: Performance Assessment of SARS-CoV-2 PCR Assays Developed by WHO Referral Laboratories
Source: J Clin Med. 2020 Jun 16;9(6):1871. doi: 10.3390/jcm9061871 (PMC7355678; doi:10.3390/jcm9061871)
Supplement: Supplementary file 1 [file jcm-09-01871-s001.pdf]

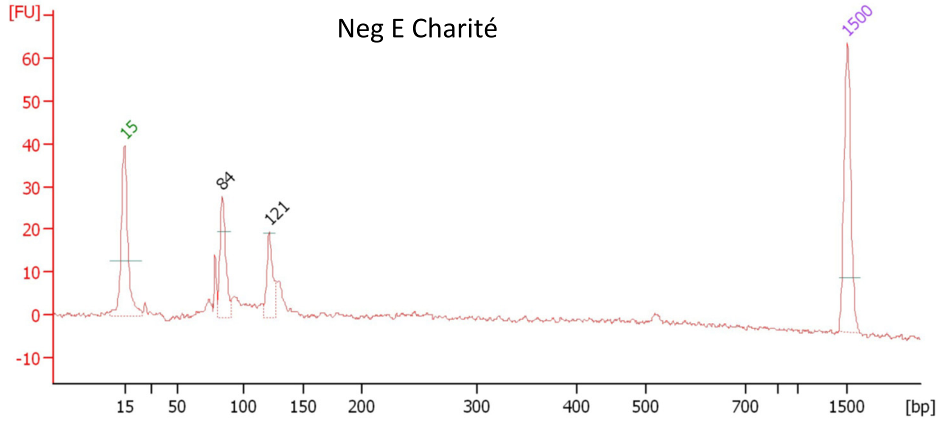
Neg E Charité
[FU]
60
50
40
30
20
10
0
-10
15
84
121
1500
15
50
100
150
200
300
400
500
700
1500
[bp]

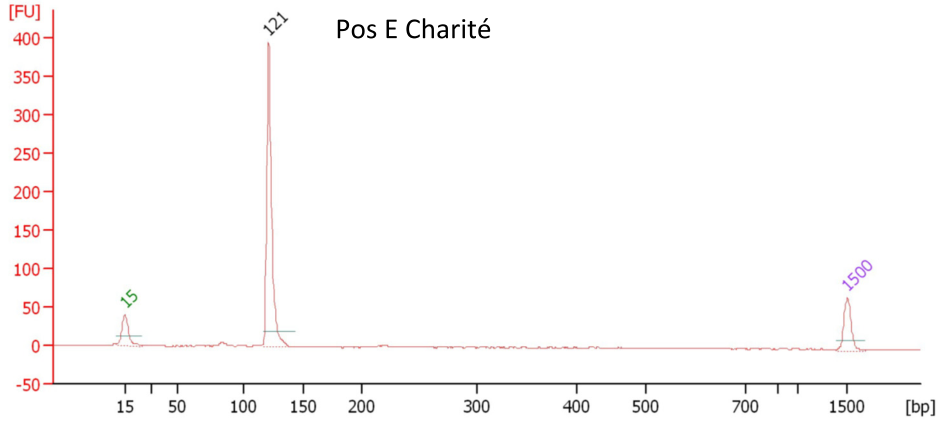
Pos E Charité
[FU]
400
350
300
250
200
150
100
50
0
-50
15
121
1500
15
50
100
150
200
300
400
500
700
1500
[bp]

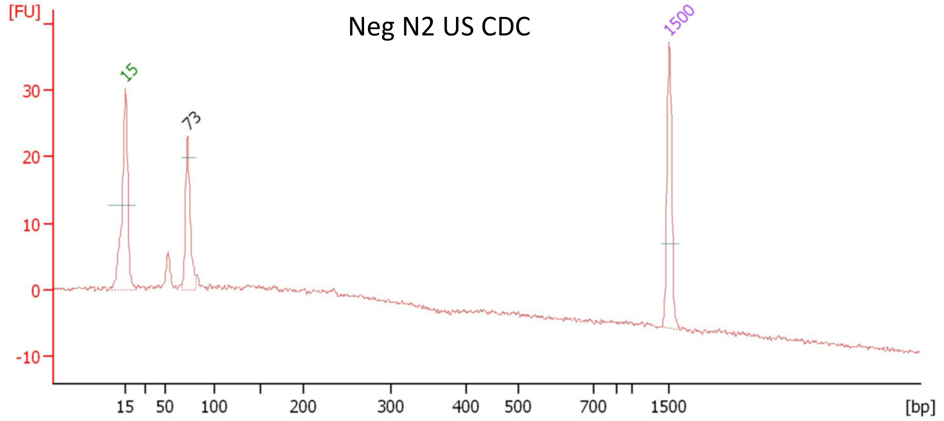
Neg N2 US CDC
[FU]
30
20
10
0
-10
15
73
1500
15
50
100
200
300
400
500
700
1500
[bp]

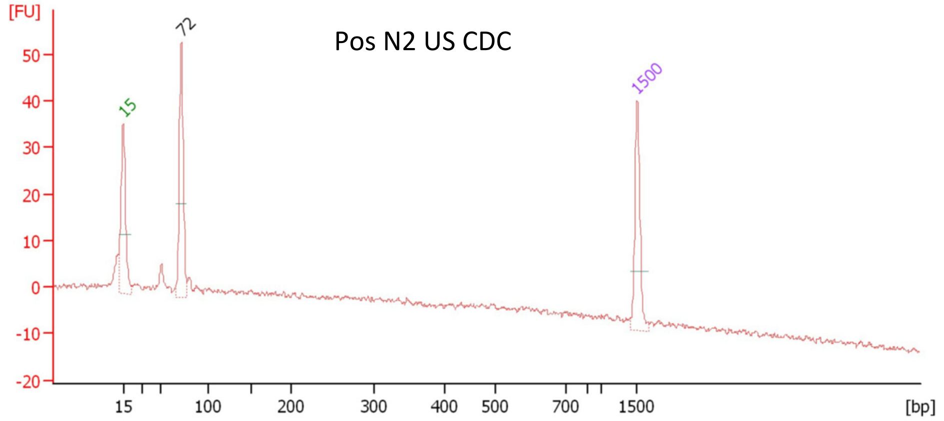
Pos N2 US CDC
[FU]
50
40
30
20
10
0
-10
-20
15
72
1500
15
100
200
300
400
500
700
1500
[bp]

Figure 1. Electrophoregrams of amplicon sizes obtained using Agilent DNA 1000 kit (Agilent technologies) for one positive sample (pos) and one negative sample (neg) for E Charité (Germany) and N2 US CDC (United States). Fluorescence unit (FU) is on y-axis and base pairs (bp) on x-axis. One lower marker (15 bp) and one upper marker (1500bp) were used.

Table 1. Cycle threshold values and mean ± standard deviation for serial dilutions of SARS-CoV-2 cell culture supernatants obtained using IP2 and IP4 Institut Pasteur, Paris (France) when used in simplex and multiplex.

	**Ct values**			
	**Institut Pasteur, Paris (France)**			
	**Simplex**		**Multiplex**	
	**Ip2**	**Ip4**	**Ip2**	**Ip4**
10^{-3}	22.3	26.6	21.0	20.9
10^{-4}	25.8	30.1	24.2	24.1
10^{-5}	28.9	33.6	28.0	27.6
	29.0	33.6	27.8	27.7
	29.1	34.5	28.1	27.8
Mean ± sd 10^{-5}	29.0 ± 0.2	33.9 ± 0.5	28.0 ± 0.2	27.7 ± 7
10^{-6}	32.1	39.1	31.5	31.2
	32.3	37.9	31.5	31.1
	32.2	37.7	31.3	31.0
Mean ± sd 10^{-6}	32.2 ± 0.1	38.2 ± 0.8	31.4 ± 0.1	31.1 ± 0.1
10^{-7}	36.5	38.6	34.3	34.3
	35.2	40.7	35.0	35.0
	35.9	38.5	34.6	34.1
	35.8	N	35.2	34.3
	38.9	46.6	34.3	33.9
Mean ± sd 10^{-7}	36.5 ± 1.4	41.1 ± 3.8	34.7 ± 0.4	34.3 ± 04
10^{-8}	39.1	ND	ND	37.0
	ND	ND	42.1	37.0
	37.1	ND	38.7	ND
	39.1	ND	ND	ND
	37.6	ND	36.2	36.1
Mean ± sd 10^{-8}	38.2 ± 1.0	NA	39.0 ± 3.0	36.7 ± 0.5
10^{-9}	ND	ND	39.9	38.8
	ND	ND	39.3	ND
	ND	ND	38.4	ND
	ND	ND	ND	ND
	ND	ND	ND	ND
Mean ± sd 10^{-9}	NA	NA	39.2 ± 0.8	NA
Water 1	ND	ND	ND	ND
Water 2	ND	ND	ND	ND

ND (non-detected) indicates a negative result for one replicate, NA (non-applicable).

Table 2. Cycle threshold values and mean ± standard deviation (sd) for serial dilutions (10^{-3} to 10^{-9}) of SARS-CoV-2 cell culture supernatants obtained using five RT-PCR assays for SARS-CoV-2 detection made available by WHO.

	Cycle threshold												
	Charité (Germany) [5,6] [5,6][5,6]				HKU (Hong-Kong) [10,11]		China CDC (China) [12]		US CDC (United-States) [13]			Institut Pasteur, Paris (France) [14]	
	**RdRp S**	**RdRp NS**	**N**	**E**	**N**	**ORF**	**N**	**ORF**	**N1**	**N2**	**N3**	**Ip2 Multiplex**	**Ip4 Multiplex**
10^{-3}	23.9	26.3	26.0	20.6	25.5	26.2	23.6	23.3	22.4	21.6	21.8	21.0	20.9
10^{-4}	27.2	28.3	29.3	23.5	28.8	29.7	27.0	27.1	25.6	25.2	25.3	24.2	24.1
10^{-5}	30.1	31.2	32.7	27.4	31.3	31.8	30.4	31.3	29.1	28.8	28.8	28.0	27.6
	29.4	31.3	32.6	27.2	31.4	31.9	30.4	31.0	29.1	28.8	28.7	27.8	27.7
	30.1	31.1	32.7	27.2	31.6	31.9	30.4	31.2	29.1	28.8	28.5	28.1	27.8
Mean ± sd 10^{-5}	29.9 ± 0.4	31.2 ± 0.1	32.7 ± 0.1	NA	31.4 ± 0.2	31.9 ± 0.1	30.4 ± 0.0	31.2 ± 0.1	29.1 ± 0.0	NA	28.7 ± 0.2	28.0 ± 0.2	27.7 ± 7
10^{-6}	33.6	34.0	35.6	30.3	35.0	35.6	33.5	35.1	32.3	32.5	31.9	31.5	31.2
	ND	33.5	35.3	31.1	35.0	34.7	33.5	35.4	32.6	32.1	31.9	31.5	31.1
	ND	33.8	35.4	30.8	35.3	35.0	33.6	34.8	32.2	32.3	31.9	31.3	31.0
Mean ± sd 10^{-6}	NA	33.8 ± 0.2	35.4 ± 0.2	NA	35.1 ± 0.2	35.1 ± 0.5	33.6 ± 0.0	35.1 ± 0.3	32.4 ± 0.2	NA	31.9 ± 0.0	31.4 ± 0.1	31.1 ± 0.1
10^{-7}	ND	ND	38.7	33.5	39.4	37.9	36.3	ND	36.0	34.4	37.1	34.3	34.3
	ND	ND	38.7	33.1	ND	38.2	36.4	39.6	36.4	34.4	34.8	35.0	35.0
	ND	ND	38.4	34.3	39.2	39.8	36.9	ND	35.4	34.4	34.5	34.6	34.1
	ND	ND	37.2	35.1	ND	ND	36.7	ND	36.0	34.2	34.5	35.2	34.3
	ND	ND	38.0	33.7	39.1	37.5	37.0	39.8	35.9	35.2	ND	34.3	33.9
Mean ± sd 10^{-7}	NA	NA	38.2 ± 0.6	NA	39.2 ± 0.2	38.4 ± 1.0	36.7 ± 0.3	39.7 ± 0.1	35.9 ± 0.4	NA	35.2 ± 1.3	34.7 ± 0.4	34.3 ± 04
10^{-8}	ND	ND	38.5	36.2	ND	ND	ND	ND	37.5	35.5	37.1	ND	37.0
	ND	ND	ND	38.0	ND	ND	39.3	ND	38.4	35.7	37.8	42.1	37.0
	ND	ND	ND	38.4	ND	ND	38.1	ND	38.0	35.0	38.4	38.7	ND
	ND	ND	39.9	ND	ND	ND	ND	ND	ND	34.9	37.5	ND	ND
	ND	ND	44.6	36.9	ND	ND	ND	ND	38.5	34.7	38.2	36.2	36.1
Mean ± sd 10^{-8}	NA	NA	41.0 ± 3.2	NA	NA	NA	38.7 ± 0.8	NA	38.1 ± 0.5	NA	37.8 ± 0.5	39.0 ± 3.0	36.7 ± 0.5
10^{-9}	ND	ND	ND	35.7	ND	ND	ND	ND	ND	35.3	38.6	39.9	38.8
	ND	ND	ND	39.7	ND	ND	39.5	ND	ND	35.3	ND	39.3	ND
	ND	ND	ND	38.5	ND	ND	ND	ND	ND	35.1	38.2	38.4	ND
	ND	ND	ND	35.5	ND	ND	ND	ND	ND	35.1	ND	ND	ND
	ND	ND	ND	36.2	ND	ND	ND	ND	41.3	35.1	ND	ND	ND
Mean ± sd 10^{-9}	NA	NA	NA	NA	NA	NA	NA	NA	NA	NA	38.4 ± 0.2	39.2 ± 0.8	NA

Water 1	ND	ND	ND	38.5	ND	ND	ND	ND	ND	35.6	ND	ND	ND
Water 2	ND	ND	ND	37.2	ND	ND	ND	ND	ND	35.5	ND	ND	ND

ND (non-detected) indicates a negative result for one replicate, NA (non-applicable).

Table 3. Cycle thresholds values obtained on four clinical samples using five RT-PCR assays for SARS-CoV-2 detection made available by WHO.

	Cycle threshold												
	Charité (Germany) [5,6]				HKU (Hong-Kong) [10,11]		China CDC (China) [12]		US CDC (United-States) [13]			Institut Pasteur, Paris (France) [14]	
	RdRp S	RdRp G	N	E	N	ORF	N	ORF	N1	N2	N3	Ip2 Simplex	Ip4 Simplex
Positive	23.3	25.4	25.1	20.7	22.5	21.6	22.4	20.9	20.5	20.5	20.4	20.0	20.4
Positive	26.8	30.1	28.8	25.4	26.3	25.7	26.6	25.2	24.9	25.0	24.4	24.5	24.6
Positive	31.3	ND	32.5	29.3	30.2	30.0	30.3	30.0	28.4	28.7	28.1	29.1	29.7
Positive	ND	ND	ND	35.0	36.8	36.2	36.0	36.1	34.5	34.1	34.4	34.5	35.8
Water 3	ND	ND	ND	36.4	ND	ND	ND	ND	ND	35.3	ND	ND	ND

ND (non-detected) indicates a negative result.

Table 4. Cycle threshold values obtained on clinical samples (n=16) using the three most sensitive methods: N China CDC, N1 US CDC, and IP2 Institut Pasteur, Paris (France).

Cycle threshold			
	**China CDC (China) [12]**	**US CDC (United-States) [13]**	Institut Pasteur, Paris (France) [14]
	N	N1	IP2 Simplex
Positive sample 5	27.76	25.79	26.88
Positive sample 6	28.75	27.28	27.24
Positive sample 7	29.1	27.12	27.74
Positive sample 8	30.41	29.09	29.14
Positive sample 9	30.71	29.37	29.38
Positive sample 10	31.14	29.32	29.51
Positive sample 11	31.03	29.1	30.15
Positive sample 12	30.85	29.72	29.81
Positive sample 13	31.41	29.58	30.06
Positive sample 14	31.86	30.46	30.87
Positive sample 15	32.4	30.52	33.07
Positive sample 16	33.29	32.82	33.17
Positive sample 17	34.03	33.35	33.35
Positive sample 18	34.33	33.19	33.51
Positive sample 19	36.19	34.36	35.36
Positive sample 20	36.82	36.11	38.68
Water 4	ND	ND	ND
Water 5	ND	ND	ND

ND (non-detected) indicates a negative result.

Table 5. Specificity assessment of five RT-PCR assays made available by WHO.

9	Cycle threshold												
	Charité (Germany) [5,6]				HKU (Hong-Kong) [10,11]		China CDC (China) [12]		US CDC (United-States) [13]			Institut Pasteur, Paris (France) [14]	
	**RdRp S**	**RdRp NS**	**N**	**E**	**N**	**ORF**	**N**	**ORF**	**N1**	**N2**	**N3**	**Ip2 Multiplex**	**Ip4 Multiplex**
Negative sample	ND	ND	ND	37.4	ND	ND	ND	ND	ND	35.7	ND	ND	ND
Negative sample	ND	ND	ND	38.3	ND	ND	ND	ND	ND	35.5	ND	ND	ND
Negative sample	ND	ND	ND	37.8	ND	ND	ND	ND	ND	35.9	ND	ND	ND
Negative sample	ND	ND	ND	35.9	ND	ND	ND	ND	ND	35.2	ND	ND	ND
Negative sample	ND	ND	ND	33.83	ND	ND	ND	ND	ND	34.35	ND	ND	ND
Negative sample	ND	ND	ND	33.04	ND	ND	ND	ND	ND	34.6	ND	ND	ND
Negative sample	ND	ND	ND	33.84	ND	ND	ND	ND	ND	33.56	ND	ND	ND
Negative sample	ND	ND	ND	33.26	ND	ND	ND	ND	ND	33.82	ND	ND	ND
Negative sample	ND	ND	ND	33.43	ND	ND	ND	ND	ND	34.17	ND	ND	ND
Negative sample	ND	ND	ND	37.03	ND	ND	ND	ND	ND	33.75	ND	ND	ND
Negative sample	ND	ND	ND	34.57	ND	ND	ND	ND	ND	34.13	ND	ND	ND
Negative sample	ND	ND	ND	33.6	ND	ND	ND	ND	ND	34.75	ND	ND	ND
Negative sample	ND	ND	ND	39.69	ND	ND	ND	ND	ND	34.07	ND	ND	ND
Negative sample	ND	ND	ND	34.71	ND	ND	ND	ND	ND	34.54	ND	ND	ND
Negative sample	ND	ND	ND	34.8	ND	ND	ND	ND	ND	34.18	ND	ND	ND
Negative sample	ND	ND	ND	38.83	ND	ND	ND	ND	ND	33.95	ND	ND	ND
Negative sample	ND	ND	ND	33.6	ND	ND	ND	ND	ND	33.67	ND	ND	ND
Negative sample	ND	ND	ND	32.65	ND	ND	ND	ND	ND	34.36	ND	ND	ND
Negative sample	ND	ND	ND	34.4	ND	ND	ND	ND	ND	33.96	ND	ND	ND
Negative sample	ND	ND	ND	33.85	ND	ND	ND	ND	ND	34.15	ND	ND	ND
Sample positive for Adenovirus	ND	ND	ND	33.1	ND	ND	ND	ND	ND	33.77	ND	ND	ND
Sample positive for Human coronavirus 229E	ND	ND	ND	33.27	ND	ND	ND	ND	ND	34.07	ND	ND	ND

Sample positive for Human coronavirus HKU1	ND	ND	ND	33.89	ND	ND	ND	ND	ND	34.65	ND	ND	ND
Sample positive for Human coronavirus HKU1	ND	ND	ND	32.8	ND	ND	ND	ND	ND	34.37	ND	ND	ND
Sample positive for Human coronavirus HKU1 and human influenza A	ND	ND	ND	33.77	ND	ND	ND	ND	ND	34.41	ND	ND	ND
Sample positive for Human coronavirus NL63	ND	ND	ND	35.12	ND	ND	ND	ND	ND	33.73	ND	ND	ND
Sample positive for Human coronavirus NL63 and human influenza A	ND	ND	ND	34.39	ND	ND	ND	ND	ND	34.35	ND	ND	ND
Sample positive for Human coronavirus NL63, human influenza B and Picornavirus	ND	ND	ND	33.65	ND	ND	ND	ND	ND	33.97	ND	ND	ND
Sample positive for Human	ND	ND	ND	34.3	ND	ND	ND	ND	ND	33.41	ND	ND	ND
Sample positive for Human	ND	ND	ND	34.29	ND	ND	ND	ND	ND	34.44	ND	ND	ND
Sample positive for Human	ND	ND	ND	37.54	ND	ND	ND	ND	ND	33.99	ND	ND	ND
Sample positive for Human influenza A and Metapneumovirus	ND	ND	ND	33.23	ND	ND	ND	ND	ND	34.35	ND	ND	ND
Sample positive for Human	ND	ND	ND	33.44	ND	ND	ND	ND	ND	34.13	ND	ND	ND
Sample positive for Human	ND	ND	ND	34.42	ND	ND	ND	ND	ND	33.8	ND	ND	ND
Sample positive for Human influenza B and Rhinovirus	ND	ND	ND	34.91	ND	ND	ND	ND	ND	33.6	ND	ND	ND
Sample positive for	ND	ND	ND	34.02	ND	ND	ND	ND	ND	33.95	ND	ND	ND
Sample positive for	ND	ND	ND	33.74	ND	ND	ND	ND	ND	34.97	ND	ND	ND
Sample positive for Metapneumovirus and Rhinovirus	ND	ND	ND	37.73	ND	ND	ND	ND	ND	34.46	ND	ND	ND
Sample positive for	ND	ND	ND	34.05	ND	ND	ND	ND	ND	36.26	ND	ND	ND
Sample positive for	ND	ND	ND	32.46	ND	ND	ND	ND	ND	35.19	ND	ND	ND
Sample positive for	ND	ND	ND	38.18	ND	ND	ND	ND	ND	34.12	ND	ND	ND

Sample positive for Parainfluenzavirus 4 and Adenovirus	ND	ND	ND	34.96	ND	ND	ND	ND	ND	34.67	ND	ND	ND
Sample positive for Picornavirus	ND	ND	ND	34.04	ND	ND	ND	ND	ND	34.23	ND	ND	ND
Sample positive for	ND	ND	ND	32.75	ND	ND	ND	ND	ND	34.21	ND	ND	ND
Sample positive for	ND	ND	ND	34.56	ND	ND	ND	ND	ND	33.94	ND	ND	ND
Sample positive for Respiratory syncytial virus and Adenovirus	ND	ND	ND	34.08	ND	ND	ND	ND	ND	34.49	ND	ND	ND
Sample positive for Respiratory syncytial virus and Rhinovirus	ND	ND	ND	33.46	ND	ND	ND	ND	ND	34.08	ND	ND	ND
Sample positive for Respiratory syncytial virus, [illegible]	ND	ND	ND	34.3	ND	ND	ND	ND	ND	34.29	ND	ND	ND
Sample positive for Rhinovirus	ND	ND	ND	33.19	ND	ND	ND	ND	ND	34.57	ND	ND	ND
Sample positive for Rhinovirus	ND	ND	ND	33.15	ND	ND	ND	ND	ND	34.54	ND	ND	ND
Water 4	ND	ND	ND	33.24	ND	ND	ND	ND	ND	34.13	ND	ND	ND
Positive control	30.83	32.39	33.35	28.52	30.79	30.4	29.21	37.97	27.14	28.09	27.84	28.22	29.97

Cycle threshold values are indicated and were obtained on clinical samples tested negative for SARS-CoV-2 (n=50) including samples tested positive for other viruses than SARS-CoV-2 (n=30) and negative samples (n=20). ND (non-detected) indicates a negative result.
